# A 5-Year Review of the Impact of Lottery Incentives on HIV-Related Services

**DOI:** 10.1007/s11904-024-00694-0

**Published:** 2024-04-04

**Authors:** Shannon Bosman, Shriya Misra, Lili Marie Flax-Nel, Alastair van Heerden, Hilton Humphries, Zaynab Essack

**Affiliations:** 1 Centre for Community Based Research, Human Sciences Research Council, Old Bus Depot, 1 Caluza Street, Sweetwaters, KwaZulu Natal South Africa; 2 SAMRC/WITS Developmental Pathways for Health Research Unit, Department of Paediatrics, School of Clinical Medicine, Faculty of Health Sciences, University of the Witwatersrand, Johannesburg, Gauteng South Africa; 3 School of Applied Human Sciences, University of KwaZulu Natal, Pietermaritzburg, South Africa; 4 School of Law, University of KwaZulu Natal, Pietermaritzburg, South Africa

**Keywords:** Lottery incentives, HIV prevention, HIV treatment, HIV testing, contextual relevance, Financial incentives

## Abstract

**Purpose of review:**

Lottery incentives are an innovative approach to encouraging HIV prevention, treatment initiation, and adherence behaviours. This paper reviews the latest research on lottery incentives’ impact on HIV-related services, and their effectiveness for motivating behaviours to improve HIV service engagement and HIV health outcomes.

**Recent findings:**

Our review of ten articles, related to lottery incentives, published between 2018 and 2023 (inclusive) shows that lottery incentives have promise for promoting HIV-related target behaviours. The review highlights that lottery incentives may be better for affecting simpler behaviours, rather than more complex ones, such as voluntary medical male circumcision. This review recommends tailoring lottery incentives, ensuring contextual-relevance, to improve the impact on HIV-related services.

**Summary:**

Lottery incentives offer tools for improving uptake of HIV-related services. The success of lottery incentives appears to be mediated by context, the value and nature of the incentives, and the complexity of the target behaviour.

## Introduction

The HIV pandemic remains a global public health challenge [1]. Despite significant progress in prevention and treatment, the UNAIDS 95–95-95 goals remain unmet [1, 2]. To achieve these global targets by 2030, innovation is needed to improve HIV testing rates, linkage to prevention and treatment services and viral suppression amongst PLHIV. Interventions using financial incentives have been used to improve engagement in HIV testing services, HIV prevention service use, and HIV treatment outcomes in different settings [3–10]. Financial incentives work by externally motivating individuals to engage in target behaviours with the hope that over time these behaviours become habitual or internally motivated [11, 12]. Given that financial barriers are frequently cited as impeding uptake of HIV car services, financial incentives may additionally serve to mitigate these [13].

Incentives are an innovative approach that can be used promote HIV-related target behaviours or outcomes [13–15]. Incentives can be offered conditionally or unconditionally depending on requirements to fulfil any behavioural conditions or not [13, 16]. Unconditional incentives indirectly improve health outcomes by making resources (e.g., food, money) more accessible [14, 16]. Conditional incentives directly motivate a target behaviour as recipients are only given incentives when they complete a particular target behaviour, with financial incentives most often used to promote behaviour change [15]. Additionally, incentives can be given as fixed incentives (equal for all) or lottery incentives (a random probability of selection for the incentive) [15].

## Lottery Incentives

Lottery incentives are attractive for public health interventions because they offer the potential benefits of financial incentive interventions at a potentially lower cost and greater ease of administration. Lottery incentives have become increasingly popular because there is compelling evidence from decades of research in applied behavioural economics showing people assign greater significance to small probabilities, preferring a small chance of a large reward than small reward for sure [15, 17–19]. The conceptual framework from Adams et al. (2014) describes how lotteries work to change behaviour [19]. Table 1 outlines the organisation of lottery incentives using this framework.

**Table 1:** Framework of domains for organisation of lottery incentives for behaviour change

Domain	Lottery incentive direction
Direction	Receiving the chance to receive a positive reward on completion of a target behaviour
Form	The nature/form of the reward (cash, vouchers or gifts) impact the strength of the reward to influence behaviour (have monetary value)
Magnitude	Variable, offering chance of winning prizes of smaller and larger value. This is affected by the reference point of the individual
Certainty	Has a chance of winning a prize with each entry but not guaranteed
Target	The type of behaviour being rewarded, complexity of the behaviour, may mediate how well the lottery works. Specific target behaviour change such as having an HIV test
Frequency	Dependant on the behaviour. May be once off for HIV test or regular chance of winning if incentivising adherence to medication
Immediacy	Entry to win a reward is received on completion of the target behaviour
Schedule	Fixed and tied to specific target behaviour such as chance of reward with each suppressed viral load result
Recipient	Provide the reward to the individual completing the targeted behaviour

Lotteries have been used to positively influence health interventions and have been employed in various settings to address HIV-related challenges, such as increasing HIV testing rates, promoting adherence to antiretroviral therapy (ART), and encouraging safer sexual practices [10, 11, 14, 20–22]. Lottery incentives could also provide more sustainable models for governments, improving health seeking behaviours, at a lower cost than unconditional and conditional incentive approaches which need to provide the reward to all who meet the conditions.

Lottery incentives have been used by governments to promote desirable health outcomes. For example, the USA used both conditional lottery and conditional cash and other incentives to nudge COVID-19 vaccine uptake [23, 24]. Cash was favoured and more effective at improving uptake. However, lottery incentives may be appealing for policymakers given that they have some benefits over conditional cash transfers: [1] They are cost-effective because they offer a reward for relatively small investment compared to CCTs where all participants receive the incentive for the desired behaviour; [2] they are easier to administer; and [3] by nature, they do not require sustainability considerations in the same way as conditional cash transfers.

It is important to understand the recent evidence for the use of lottery incentives for the promotion of HIV-related services. This review explores evidence, from the last five years, on the impact of lottery incentives on HIV testing, prevention, and treatment interventions. Additionally, we aim to comment on the findings and make evidence-based recommendations to optimise these innovations.

## Methodology

**Fig. 1:**
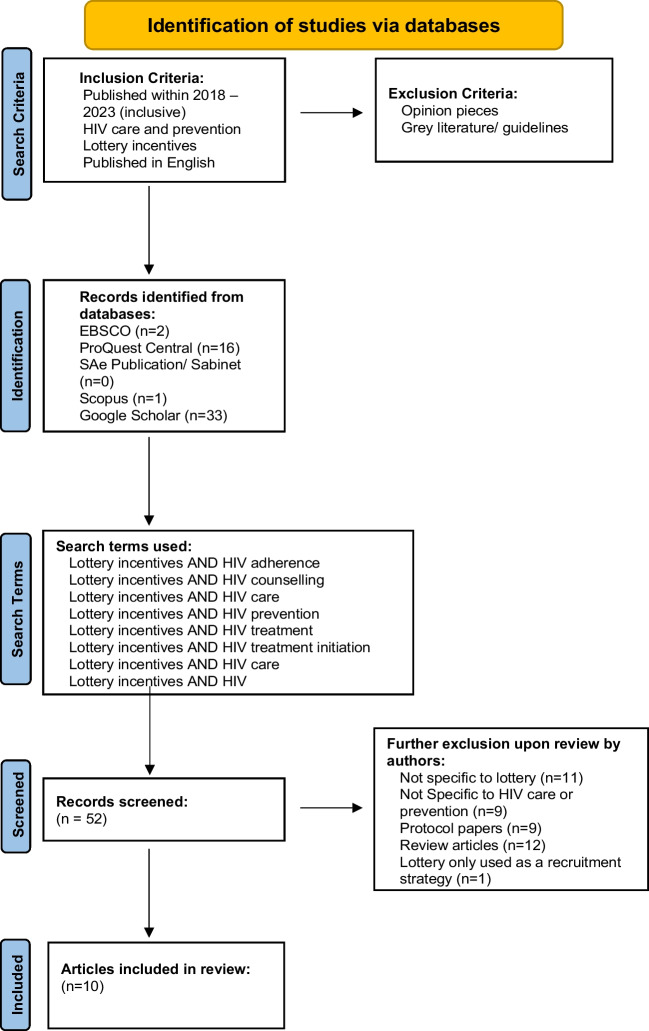
PRISMA flow diagram

This scoping review of articles published between 2018 and 2023 (inclusive) utilised the Preferred Reporting Items for Systematic Reviews and Meta-Analyses (PRISMA) outline, based on the original methodology put forward by Arksey and O’Malley [25, 26]. Figure 1 below shows the PRISMA flow, outlining the search databases and search terms used, and the relevant screening procedures completed for inclusion. Ten published articles are included in this review.

## Findings

This review found that lottery incentives have variable effects on target behaviours depending on several factors, such as the context in which the incentive was being offered, the target population, and the incentivised behaviour. To provide greater insight into the findings, this section has been divided into three main categories: the effects of lottery incentives on HIV testing-, HIV prevention-, and HIV treatment services.

### The Effects of Lottery Based Incentives on HIV Testing Services

**Fig. 2:**
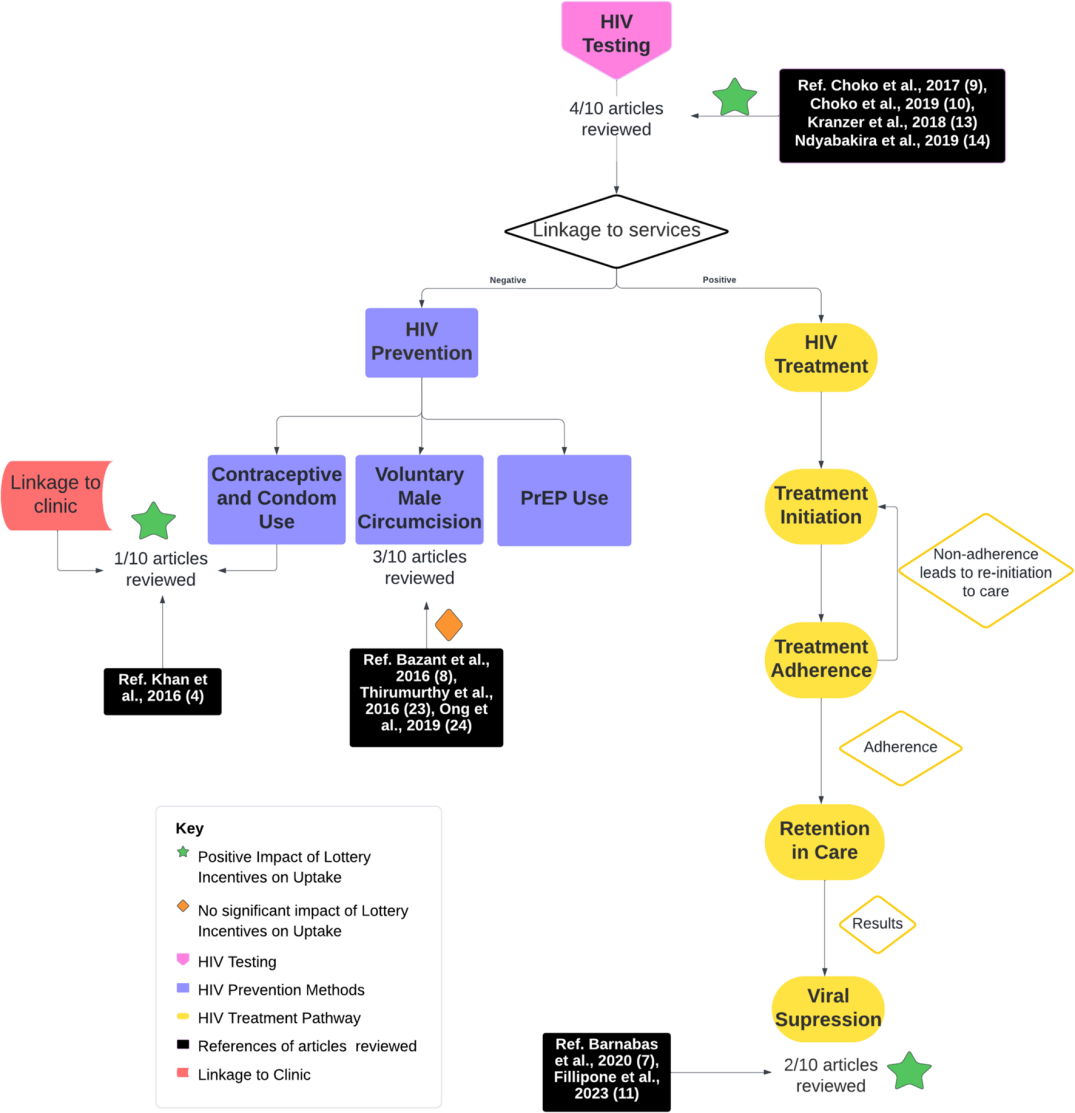
HIV-related services and the potential areas of impact for lottery incentives

HIV testing is the first critical step to achieving the UNAIDS 95–95-95 targets. Incentivising HIV testing addresses the first 95 target and may support improved linkage to HIV prevention and treatment services. As seen in Fig. 2, four articles were reviewed that explored lottery incentives as an intervention to improve the uptake of HIV testing [5, 6, 9, 10].

A randomised control trial (RCT) in urban Malawi assessed the effect of financial incentives on the secondary distribution of HIV self-test (HIVST) kits to men through their pregnant partners attending antenatal care (ANC) [6]. A sample size of 2349 pregnant women and their partners were randomised into one of the following arms: the standard of care (an invitation to male partners for clinic-based HIV testing), HIVST alone, and HIVST coupled with three financial incentive conditions: $3 or $10 fixed incentive or an entry to a $30 lottery [6]. Women who attended ANC for the first time were given the relevant referral/HIVST to give to their partners who then needed to return to the clinic for post-test counselling services within 28 days [6]. The lottery arm was dropped at interim analysis because no difference was observed between HIVST with lottery and HIVST alone [6]. However, the HIVST coupled with fixed incentives increased the uptake of post-test counselling services among the male partners, with the $10 incentives having a greater effect [6]. Various factors may affect how incentives are perceived by their intended beneficiaries. Formative qualitative work conducted before the RCT, highlighted important factors that may have mediated the acceptability of incentives in this intervention [5]. Feedback regarding lottery incentives was largely negative; they were regarded as unfair, and there was concern that participants who did not win would dissuade others from attending the clinic [5]. Fixed incentives, however, were perceived to compensate for the costs of clinic attendance [5]. Taken together with the results from the RCT, these data suggest that fixed incentives are preferable to lottery incentives in low economic settings as they better address the financial barriers to service uptake.

A study comprising 2050 households in Harare, Zimbabwe, examined the use of financial incentives to improve the uptake of clinic-based HIV testing for children and adolescents [8–17 years old] [9]. This study evaluated the effect of no incentives, fixed incentives ($2), and lottery incentives ($5 or $10) on uptake of referral for HIV testing. The rates of HIV testing significantly increased with both fixed incentives (48%) and lottery incentives (40%) as compared to the control group (20%); however, there was no statistical difference between the two incentivised groups [9]. In this study, both fixed and lottery incentives were sufficient to encourage caregivers to take their children to the clinic for HIV testing. [9] These interventions may be bolstered by incentivising both the children and the caregivers. Incentivising caregivers only could raise ethical concerns, such as the possibility of caregivers pressuring their children to participate. [9] Lottery incentives, however, may serve as a means of overcoming this concern, as they are not guaranteed. [9].

A qualitative study, in rural Uganda, examined men's perceptions of using lottery incentives to improve the uptake of community-based HIV testing. [10] Sixty in-depth interviews were conducted with purposefully sampled men (age, incentive group and campaigns attended). Similar to findings of the other qualitative study reported above [5], participants noted that incentives may offset associated costs of testing, such as loss of wages. However, participants conveyed that testing should be convenient but that incentives alone were insufficient to overcome anticipated stigma associated with testing. There were concerns about whether lottery incentives were real and could be won. Observing others winning the lottery for testing served as “social proof”, that lottery prizes were winnable. When the prize was perceived to be of low value, the lottery did not motivate the uptake of testing services. Where participants had already decided to test for HIV, lottery incentives served as a “cue to action” and “sweetened the deal”, acting as a secondary motivator. Lottery incentives may serve as an added motivation for HIV testing uptake and offer an offset of financial barriers.

The use of fixed and lottery incentives promotes uptake of HIV testing, as noted in Fig. 2 (green star). While the use of lottery incentives to enhance the uptake of HIV testing services may serve as an added motivation [5, 6, 9, 10], and has increased the uptake of testing in children and adolescents, it has not proven beneficial in increasing men attending the clinic for post-HIVST counselling services. A well-designed lottery incentive intervention can be useful in promoting the intended target behaviour.

### The Impact of Lottery based incentives on HIV Prevention Services

Figure 2 above depicts the HIV prevention strategies (four articles) that examined the use of lottery incentives, aimed to motivate prevention behaviours. These prevention strategies included voluntary medical male circumcision (VMMC) and condom use. [4, 8, 27, 28] The use of oral pre-exposure prophylaxis (PrEP) is an effective prevention tool and has been incentivised using fixed financial incentives. [29] This review found no examples of lottery incentives being used to promote PrEP.

#### Voluntary Medical Male Circumcision

Three articles explored the use of lottery incentives in promoting uptake of VMMC. [4, 27, 28] Two articles reported findings from RCTs, which showed that lottery incentives did not significantly increase the uptake of VMMC. In an RCT conducted in Tanzania, lottery incentives were offered to participants contingent on their uptake of VMMC. There was a greater increase in the VMMC uptake in lottery intervention clinics (47%) than in the control group (8%), however, this increase was not statistically significant, shown in Fig. 2 (orange diamond). Smartphones were offered as the incentive​, which participants reported to be irrelevant to their setting – where there was no electricity. The nature of the incentive is a critical consideration, and in this context small cash incentives or transport reimbursement were preferred. [4].

An RCT conducted in Kenya reported that fixed incentives were found to significantly increase VMMC uptake, compared to lottery and control groups. [4, 27] There was no statistical difference in the uptake of VMMC between the control and lottery groups. Fixed incentives offered $12.50 food voucher to all participants, lottery incentives offered a variety of prizes and probabilities, and the control group offered $0.60 cash. All incentives were contingent on having a VMMC done within three months. [27] Lottery incentive intervention offered a certainty of winning; with an 85% chance of receiving a $2.50 food voucher, 10% chance of winning a $45 standard phone or pair of shoes, and a 5% chance of winning a $120 bicycle or smartphone. [27] Both the fixed and lottery incentive groups had a certainty of winning a prize, with varied values. The minimum lottery incentive ($2.50) may have been inadequate to overcome physical, financial and psycho-social barriers to VMMC and may explain why fixed incentives were more effective. [27, 30, 31].

The third article described a discrete choice experiment conducted with 325 adult men in Tanzania, exploring preferences for VMMC. Men were divided into two groups by latent class analysis to test the hypothesis that men who engaged in more risky sexual activity would find lottery incentives more acceptable. An overwhelming majority of men (84%) showed a preference for transport vouchers over lottery incentives. [28] Where the behaviours are more complex (e.g., VMMC), participants may require a more definite form of compensation, such as fixed incentive or alternatively, a higher value lottery.

These findings highlight the importance of conducting formative research when designing lottery interventions to ensure these incentives are relevant and are sufficiently valuable to encourage target behaviours.

#### Condom Use

Lottery incentives have been tested for improving dual protection (contraception and condom use) amongst women, in Cape Town, South Africa. [8] Participants were incentivised to achieve an STI-free status by using condoms. At the six-month post enrolment visit participants were tested for STIs, and those who were negative received a lottery entry. The study found that lottery incentives increased the odds of returning to the clinic, as seen on Fig. 2 (orange diamond) and the uptake of dual protection which resulted in fewer STIs in the lottery group as compared to the control. [8].

These findings suggest that the nature of the target behaviour being incentivised is important. Complex behaviours such as VMMC may not be appropriate for lottery interventions, whereas comparatively simpler behaviours such as condom usage are strengthened by lottery incentives. While condom use does require partner negotiations and navigating complex social issues, it has fewer mitigating factors than undergoing VMMC which is a surgical procedure.

### The Impact of Lottery Based Incentives on HIV Treatment

Treatment initiation, adherence and retention in care are vital for attaining viral suppression which in turn prevents onward transmission, disease progression and viral resistance; critical to achieving the UNAIDS 95–95-95 goals. [1, 2] A South African study showed that, following HIV testing, lottery incentives decreased the time it took for participants to register at a local clinic, but did not influence the rate of ART uptake between the intervention and control groups. [3] This same study also showed an increase in rates of viral suppression among participants who received lottery incentives, as seen in Fig. 2 (green star), but the viral suppression phase of the study was underpowered to make any definitive conclusions. [3] However, the improvement in linkage to care is significant, as the sooner PLHIV are initiated on ART, the sooner they can achieve viral suppression, through maintained adherence.

A USA-based study showed that lottery incentives improved the rates of ART adherence, among HIV-positive men and women. [7] Participants were incentivised to adhere to their treatment by receiving a fixed or lottery incentive when they tested virally suppressed at four- and eight-month follow-up visits. While both fixed ($300) and lottery incentive groups showed an increase in viral suppression, the lottery incentive was significantly more effective. Interestingly, this lottery was set up in such a manner that all participants were guaranteed to win a prize, the only variable was the amount of money they received (70% chance of winning $250 and 30% chance of winning $500). [7].

This is significant because receiving a prize was guaranteed. However, a guaranteed prize in and of itself is insufficient to encourage target behaviours as shown above with the VMMC study conducted in Kenya. Key differences with the intervention include the much higher value of the incentives and the comparative difference between the value of lottery and incentives. Considering that this was the only one of ten studies that was conducted in the USA, further research is needed to see if these results translate into a Sub-Saharan African LMIC context where HIV is most prevalent.

Although ART is a lifelong treatment, incentive-based interventions are only offered for a short period of time with the intention of building a habit that would sustain the behaviour change beyond the intervention period. In the USA-based study, participants expressed their commitment to maintaining medication adherence even after the intervention concluded (i.e., once incentives have stopped), but more information is needed to assess sustainability of treatment adherence. [7] Additional data is required to determine the optimal duration for providing incentives to encourage ART adherence. Research is needed to evaluate how the length of time for which the incentive is offered, affects this habit formation and the applicability across contexts, as well as the sustainability of lottery incentives as a public health intervention.

Figure 2 outlines the articles included in this review, which indicate the promise of lottery incentives, in improving linkage to care and motivating treatment adherence. While lottery intervention shows promise in supporting viral suppression in the short term, more data is needed to assess the long-term effects, particularly in Sub-Saharan African contexts.

## Conclusion

In efforts to end the global HIV pandemic by 2030, lottery incentives may provide an innovative tool to promote HIV prevention, treatment and care behaviours. To this end, several studies have evaluated the acceptability and efficacy of lottery incentives for HIV-related target behaviours.

This review of recent literature explored the impact of lottery incentives on HIV-related services. Together, data form the ten articles included in this analysis provide inconsistent evidence for the effectiveness of lottery incentives in promoting HIV-related service uptake. The success of lottery incentives appears to be mediated by context, the value and nature of the prize, and the complexity of the target behaviour.

The review highlights that lottery incentives may be less impactful when used to target complex behaviours that require continued intention to action or are reliant on the co-operation of others. Lottery incentives were most successful in discrete behaviours like HIV testing or improving clinic attendance and reported use of condoms by men. However, they were less effective in supporting daily adherence to medication which is an ongoing action requiring sustained behaviour change and this behaviour change may not persist once the chance of the reward is removed. Lottery incentives offer a more affordable intervention than fixed cash incentives and could be leveraged to change discrete health behaviours, such as seeking an HIV test, and support initial entry into the HIV care cascade, as shown by improved linkage to care.

Within this review, the following recommendations were made.

### Recommendations

1. This review shows that lottery incentives have a significant effect in promoting or improving healthcare provider HIV testing rates, but not on the secondary distribution of HIV self-tests. Lottery incentives were found to be acceptable as a means of motivating both self-testing and community-based testing, as well as HIV testing amongst children and adolescents. More research is needed to fully understand the effect of lottery incentives on HIV testing.
2. Since VMMC is a complex and invasive procedure, it was found that lottery incentives may not be as effective, as fixed incentives for this type of target behaviour. We recommend fixed incentives for motivation for VMMC uptake.
3. Lottery incentives show promise as an intervention for improving condom use. Our review showed that condom use, measured by an STI-free status, was improved using lottery incentives.
4. More research is needed to evaluate the effect of lottery interventions on other prevention methods such as oral PrEP and other new HIV prevention technologies as they become available.
5. Lottery incentives showed promise in promoting linkage to care and motivating treatment adherence and achieving viral suppression. More research is needed to optimise the time for which incentives are offered and their sustainability as a public health intervention in an African context.
6. These data show that incentives that are not contextually relevant are less effective. This underpins the importance of conducting formative research when designing lottery interventions.

Overall, lottery incentives have the potential to make an impact on some areas of HIV prevention and treatment. Their design and implementation should be tailored to be specific contexts, considering the recommendations made above. Further research and thoughtful intervention design can help maximize the potential benefits of lottery incentive approaches in the fight against HIV, contributing to ending the pandemic and achieving the UNAIDS 95–95-95 targets by 2030.
